# Application of High-Throughput Sequencing on the Chinese Herbal Medicine for the Data-Mining of the Bioactive Compounds

**DOI:** 10.3389/fpls.2022.900035

**Published:** 2022-07-14

**Authors:** Xiaoyan Liu, Xun Gong, Yi Liu, Junlin Liu, Hantao Zhang, Sen Qiao, Gang Li, Min Tang

**Affiliations:** ^1^School of Life Sciences, Jiangsu University, Zhenjiang, China; ^2^Department of Rheumatology and Immunology, Affiliated Hospital of Jiangsu University, Zhenjiang, China; ^3^Institute of Animal Husbandry, Jiangsu Academy of Agricultural Sciences, Nanjing, China; ^4^Department of Vascular Surgery, The Second Affiliated Hospital of Shandong First Medical University, Taian, China

**Keywords:** Chinese herbal medicine, high-throughput sequencing technologies, genome assembly, bioactive compounds, functional genes

## Abstract

The Chinese Herbal Medicine (CHM) has been used worldwide in clinic to treat the vast majority of human diseases, and the healing effect is remarkable. However, the functional components and the corresponding pharmacological mechanism of the herbs are unclear. As one of the main means, the high-throughput sequencing (HTS) technologies have been employed to discover and parse the active ingredients of CHM. Moreover, a tremendous amount of effort is made to uncover the pharmacodynamic genes associated with the synthesis of active substances. Here, based on the genome-assembly and the downstream bioinformatics analysis, we present a comprehensive summary of the application of HTS on CHM for the synthesis pathways of active ingredients from two aspects: active ingredient properties and disease classification, which are important for pharmacological, herb molecular breeding, and synthetic biology studies.

## Introduction

The Chinese herbal medicine (CHM) makes a great contribution to the human healthcare and clinical therapy due to its remarkable efficacy and fewer side effects (Flower et al., 2012; Lu et al., 2019; Xiang et al., 2019; Zheng et al., 2020). Since the Qin and Han dynasties, the Chinese ancestors had made the natural plants to cure patients without the knowledge of the chemical constituents, which gradually formed the later mature system of CHM with clarified the properties (He et al., 2015). *Artemisia annua*, an ancient medicine, grows broadly in China, the province such as, Jiangsu, Shanxi, Guangdong. Artemisinin, the main medicinal ingredient of *A. annua*, is world-famous for its treatment of malaria. Moreover, both traditional and modern pharmaceutical research imply it has anti-inflammatory, anti-viral, and anti-cancer effects (Nair et al., 2019; Feng et al., 2020; Liu H. et al., 2021). Accumulated evidence suggests that the bioactive components originated from CHM play a non-negligible role in the treatment of diseases (Li et al., 2016; Ji et al., 2019). However, the low-abundance is insufficient to meet the clinical requirements, such as paclitaxel, a well-known natural anti-cancer drug (Sabzehzari et al., 2020; Xiong et al., 2021). Nowadays, with the continuous advances in sequencing technologies for the fine genome assembly, the synthesis of bioactive components, or called cell-Bio-fluid sync, can be well elucidated, which provides abundant genetic resources for life and pharmaceutical sciences.

Direct application of the conventional Sanger sequencing (called first-generation sequencing) on the herbs with large and complicated genomes is grudging because of the low throughput and accuracy (Lo and Shaw, 2019). Instead of it, the next-generation sequencing (NGS, also called second-generation sequencing) was gradually applied since 2010 (Cheng Q. et al., 2021). NGS can perform millions to billions of independent sequencing processes, dramatically increasing sequence output, which includes Illumina Solexa, Roche454, and ABI SOLiD platform. Based on the principle of reversible termination and fluorescently labeled dNTP, Illumina Solexa is sequencing while synthesizing (Guo et al., 2021a). It also has certain drawbacks, such as short read length (usually 200-800 bp), base mismatches, GC preference, and template migration during PCR amplification, which might influence the accuracy and integrity of sequencing data (Cheng Q. et al., 2021; Guo et al., 2021a). Subsequently, the third-generation sequencing (TGS) stands out for the high-throughput sequencing (HTS) technologies as a routine method. Oxford Nanopore and PacBio single-molecule real-time (SMRT) sequencing technology are now the main TGS platforms. Although SMRT can achieve read lengths of 100 kb, additional factors like template breakage, enzyme denaturation, and short library sequences can affect read lengths and accuracy. Unlike SMART sequencing, the Nanopore read length is determined by the length of the DNA molecules to be sequenced rather than the sequencing technique (van Dijk et al., 2018). Here, we mainly reviewed relevant articles from the recent decades and presented a comprehensive summary of the application of HTS on CHM for the synthesis pathways of active ingredients from two aspects: active ingredient properties and disease classification.

## *De novo* Genome Assembly of Herbs and Bioinformatics Analysis

**Figure 1:**
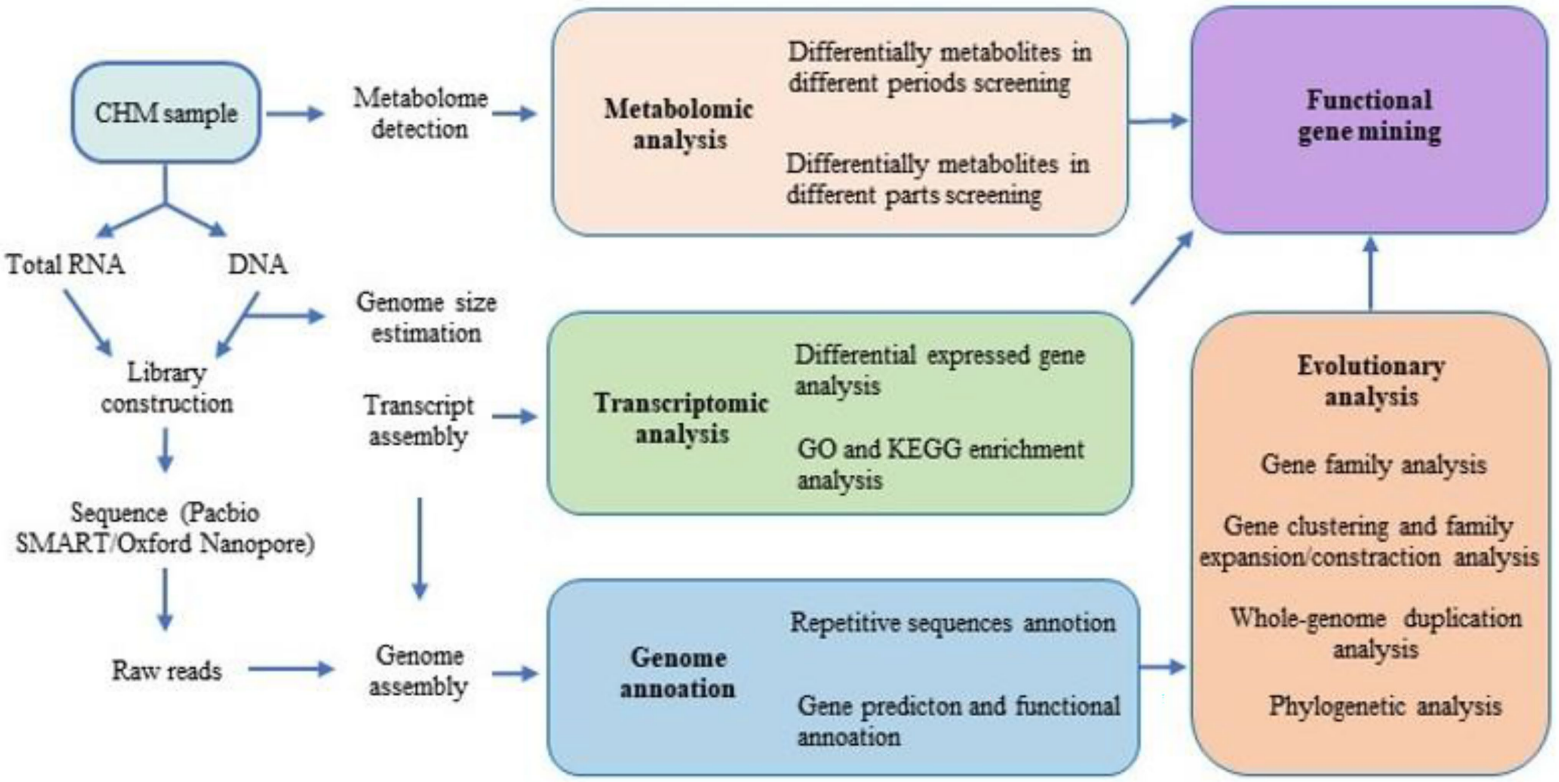
Flow chart of bioactive compound related functional genes discovering based on high-throughput sequencing (HTS).

The last 5 years have been an era of expansion in medicinal plants genome sequencing, with 2nd and 3rd generation technologies combining to assure long read length, high throughput, and reasonable sequencing price for medicinal plant genomes assembled to the chromosome level. The employment of HTS technologies on data-mining bioactive compounds is shown in Figure 1. This strategy integrates genomics, transcriptomics, and metabolomics data to analyze genomic properties, synthetic and metabolic pathways of bioactive constituents, the overall transcriptional activity of organisms, and pathway regulatory mechanisms that will be revealed to uncover functional genes (see Table 1). The *Panax notoginseng* genome, for example, has been assembled in five versions. Chen et al. (2017) and Zhang D. et al. (2017) used Illumina technology to assemble a sketch of the *P. notoginseng* genome, but the assembly was highly fragmented. Fan G. et al. (2020) employed Pacbio and Oxford Nanopore technologies to assemble the genome to the chromosome level in 2020, with significantly improved assembly continuity. The last two versions were assembled more accurately by using a combination of 2nd and 3rd generation sequencing (Jiang Z. et al., 2021; Yang Z. et al., 2021). In contrast to the study by Jiang Z. et al. (2021) and Yang Z. et al. (2021) illustrated dencichine biosynthesis, the other major bioactive compounds derived from *P. notoginseng*. *Vaccinium darrowi*, the diploid blueberry, benefits cardiovascular, neural, and retinal. Cui et al. (2022) obtained a *de novo* genome assembly for *V. darrowi* according to Oxford Nanopore, Illumina short reads, and Hi-C data. *Sapindus mukorossi*, an environmentally herb, has been used for treating inflammatory conditions owing to its abundant active compounds. Xue et al. (2022) revealed the first reference genome sequence of *S. mukorossi*. Li et al. (2022) assembled the high-quality reference genome of *Gentiana dahurica* using Nanopore long reads, Illumina short reads, and Hi-C technologies, which is the first chromosome-level genome of Gentianaceae. Based on comparative genomic and transcriptome analyses, *cytochrome P450* candidate genes related to gentiopicroside biosynthesis were identified. Noteworthy, Wang et al. (2022) employed genome-wide association studies (GWAS) and identified six quantitative trait loci (QTLs) related to fruit traits according to the latest version genome of *Dimocarpus longan* at the chromosome level with 455.5 Mb assembled into 15 chromosomes. Based on the genome-assembly and the downstream bioinformatics analysis, these articles weremainly focusing on providing new insight for the discovery of novel drug candidates in CHM.

**Table 1:** Summary of the bioactive compounds related functional genes from the assembly Chinese herbal medicines (CHM) genome.

Name	Chinese name	Functional genes	Validation methods	Download*	References
*Coptis chinensis*	黄连	*Cch00017825*	Enzymatic assays	PRJNA662860	Chen D. et al., 2021; Liu Y. et al., 2021
*Panax ginseng*	人参	*UGT94Q2*, *HMGRs*	/	PRJNA385956	Chen et al., 2017; Xu et al., 2017; Zhang D. et al., 2017; Xue et al., 2019; Fan G. et al., 2020; Zhang et al., 2020; Jiang Z. et al., 2021; Yang Z. et al., 2021
*Taxus chinensis*	红豆杉	*CYP725As*	Enzymatic assays, qRT-PCR	PRJNA730337	Xiong et al., 2021
*Tripterygium wilfordii*	雷剬藤	*CYP728B70*	Construct RNAi lines	PRJNA542587	Tu et al., 2020
*Dioscorea zingiberensis*	山药	*CYP90G6*, *CYP94D144*	Metabolic engineering in yeast	PRJNA541739	Cheng J. et al., 2021
*Salvia miltiorrhiza*	丹参	*CYP71D375*	Construct RNAi lines, enzymatic assays	PRJNA682867	Ma Y. et al., 2021
*Carthamus tinctorius*	红花	*CtAH08G60157600*	/	PRJNA642978	Wu et al., 2021
*Lithospermum erythrorhizon*	紫草	*PGT1*	Construct RNAi lines	PRJNA596998	Auber et al., 2020
*Senna tora*	决明	*CHS-L9*	Enzymatic assays	PRJNA605066	Kang et al., 2020b
*Dendrobium officinale*	铁皮石斛	*CYP71D*, *CYP75A*, *CYP75B*, *MWL1*	/	PRJNA662181	Niu et al., 2021
*Glycyrrhiza uralensis*	甘草	*UGT73B1*, *CYP72A9*	/	PRJDB3943	Mochida et al., 2017
*Platycodon grandiflorus*	桔梗	*CYP716A*, *bAS*	/	PRJNA656905	Kim et al., 2020; Yu H. et al., 2021
*Artemisia annua*	青蒿	*HMGR*(AA201470), *FPS*(AA043570, AA174930), *DBR2*(AA04970,AA049710)	Construct the overexpressing transgenic lines	PRJNA416223	Shen et al., 2018
*Cannabis sativa*	大麻	*THCAS*, *CBDAS*	/	PRJNA562042	van Bakel et al., 2011; Hurgobin et al., 2021
*Ophiorrhiza pumila*	短小蛇根草	*OG0015245*	/	PRJDB8685	Rai et al., 2021
*Zingiber officinale*	生姜	*C3OMT2*, *C3OMT3*, *C3OMT13*	/	PRJNA647255	Li H. et al., 2021
*Camptotheca acuminata*	喜树	*CarGene13172*, *CarGene10888*	/	PRJNA639006	Kang et al., 2021
*Andrographis paniculata*	穿心莲	*UGT73AU1*	Enzymatic assays	PRJNA549104	Li L. et al., 2017, 2019; Sun et al., 2019; Yue et al., 2019

^*^The fifth column is the Bioproject accession number in the NCBI database.

## Application of High Throughput Sequencing in Bioactive Compounds Discovery

**Figure 2:**
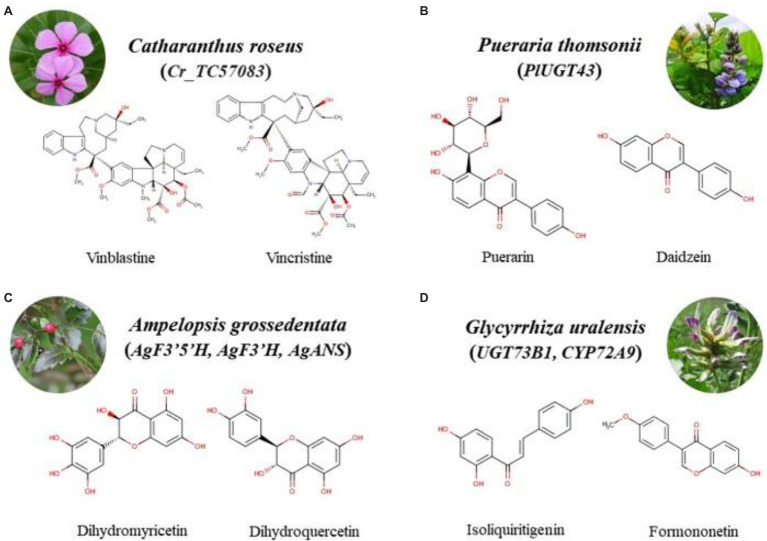
Chemical structure formula of the bioactive compounds from four species. ^*^Bioactive compound-related functional genes are shown in the brackets. **(A)** Cr_TC57083, the functional gene related to synthesis of vinblastine and vincristine in *Catharanthus roseus*; **(B)** PlUGT43, the functional gene involved in synthesis of puerarin and daidzein in *Pueraria thomsonii*; **(C)** AgF3’5’H, AgF3’H, AgANS, the functional genes associated with biosynthesis of dihydromyricetin and dihydroquercetin in *Ampelopsis grossdentata*; **(D)** UGT73B1, CYP72A9, the functional genes correlation with synthesis of isoliquiritigrnin and formononetin in *Glycyrrhiza uralensis*.

Based on the accumulation in the sequencing field, many useful bioactive compounds and their varieties have been screened out from the complex mixtures and the clinic effects have been validated (see Figure 2; Supplementary Figure S1). Specially, the secondary metabolites constitute the backbone of many drugs as the active ingredients of the medicinal plants and are widely used in pharmaceutical products. In recent years, due to the innovation of sequencing technology, the HTS accelerates the study of secondary metabolites biosynthetic in the medicinal plants, which indirectly expands the global commercial market of the herb products (Barbosa et al., 2019). In general, the secondary metabolites are divided into seven major groups, namely flavonoid, terpenoid, alkaloid, phenylpropanoid, quinone, tannin, and steroid (Lo and Shaw, 2019; Erb and Kliebenstein, 2020).

### Terpenoids

Terpenoids are hydrocarbon compounds consisting of isoprenoid as structural units (Chen et al., 2011). There are two synthetic pathways for terpenoid in medicinal plants: MVA pathway and DOXP/MEP pathway. In the MVA pathway, IPP is condensed to generate DAMP by IDI, and FDPS converts IPP and DMAPP to FPP, which are the common precursors of sesquiterpenes, triterpene, and sterol (Lichtenthaler, 1999). Monoterpene, diterpene, and tetraterpene produce in the MEP pathway (Rohmer, 1999). The first rate-limiting enzyme in the MVA pathway, 3-hydroxy-3-methylglutaryl-CoA reductase (HMGR), is critical for its regulation (Korth et al., 1997). Cytochrome P450 hydroxylases (CYP450) are used to structurally modify terpene end-products to eventually become a large number of natural products. Tu et al. (2020) reported a reference genome of *Tripterygium wilfordii* using PacBio long reads, 10X Genomics, and high throughput chromosome conformation capture (Hi-C) data and annotated 28,321 protein-coding genes. Gene Ontology (GO) and Kyoto Encyclopedia of Genes and Genomes (KEGG) analyses showed that 951 *T. wilfordii*-specific genes were especially enriched in terpene synthases. According to the gene-to-metabolite network, 57 *CYP* genes may be involved in the triptolide biosynthesis pathway were identified, of which 10 candidate genes with tissue-specific were selected for further functional validation. RNAi studies found that the transcript levels of four candidate genes (CYP728B70, TW011445.1, TW012149.1, and TW006625.1) were decreased and triptolide accumulation showed the same results. Additionally, based on substrate feeding and overexpression studies, the findings showed that CYP728B70 was involved in triptolide biosynthesis. This finding provides new insight into triptolide biosynthesis and the theoretical foundation for heterologous bioproduction (Tu et al., 2020). Xu et al. (2017) used the Illumina Hiseq X-Ten platform to sequence the genome and the transcriptome of *Panax ginseng* and predicted 42,006 protein-coding genes. Weighted gene coexpression network analysis (WGCNA) obtained 15,762 genes positively associated with ginsenosides, which are produced by the precursor IPP *via* the MVA pathway. BLAST search discovered 31 genes were linked to 10 upstream enzymes. Finally, eight genes encoding HMGRs found that they may perform different roles during ginseng development. This is of great significance for future studies on ginseng breeding and synthetic biology. Xia et al. (2018) reported a high-quality draft genome of *Siraitia grosvenorii* using SMRT sequencing *via* PacBio platform and Illumina paired-end reads. According to the genomic information, 127 candidate genes were found in the mogrosides biosynthesis pathway, including SQEs, EPHs, CYP450s, and UGTs. In addition, mogrosides are accumulated during the development of *S. grosvenorii* fruit. Up-regulated genes in fruit development were found significantly enriched in the sesquiterpenoid and triterpenoid biosynthesis pathways through RNA-seq data and KEGG analysis. This study for *S. grosvenorii* genome assembly and annotation will contribute to the discovery of new functional genes. Chen et al. performed PacBio sequencing technologies to construct the first full-length transcriptome of *Pogostemon cablin* and annotated 102 transcripts related to patchoulol biosynthesis. Patchoulo, the main bioactive compounds, among the 39 chemical compositions in *P. cablin* were detected by GS-MS analysis. Patchoulol synthase (*PatPTS*) converts famesy1-pp (FPP) to patchouli alcohol (PA). Furthermore, based on the *P. cablin* full-length transcriptome and transcriptome profiling under MeJA treatment, 427 DEGs were up-regulated in leaves after MeJA treatment, of which *HMGR*, *DXS*, *HDR*, *IDI*, *FDPS*, *PatPTS* genes related to patchouli biosynthesis were up-regulated under MeJA treatment and positively correlated with patchouli content. Although this study did not validate the identified genes using qRT-PCR, it provides a valuable genetic resource for further research in patchouli. Dong et al. (2021) constructed the genome of *Magnolia biondii* using SMRT *via* PacBio long read, 10X Genomics and Hi-C data. The chromosome-level reference genome of *M. biondii* is approximately 2.22 Gb long and predicted 47,547 protein-coding genes. The volatile oil extracted from the flower buds of *M. biondii* has many pharmacological properties such as anti-inflammatory and is rich in terpenoids, which are catalyzed by terpene synthase (TPS). Based on genomic information and RNA-seq data, 102 TPS genes were identified and the expression profiles showed 33 TPS genes were higher expressed in flowers than in leaves. These findings will improve the understanding of the molecular breeding of *M. biondii*. Kim et al. (2020) assembled a draft genome of *Platycodon grandiflorus* through PacBio platform and contained 40,017 protein-coding genes. Gene family expansion and contraction analysis found that CYP76C, CYP72, and CYP716 families in *P. grandiflorus* underwent expansion. Based on orthologous gene annotation, *β-amyrin synthases* (*bASs*) were found that underwent expansion in *P. grandifloras.* Previous research has revealed that the CYP716 gene family was involved in the platycodon saponins biosynthesis. Additionally, among the expanded gene families, *CYP716* and *bAS* genes were highest expression in roots than other tissues. To investigate the terpenoid biosynthesis pathway in *Artemisia argyi*, gene expression analysis was performed and found 36,820 non-redundant transcripts, of which 187 transcripts relevant to terpenoid biosynthesis were discovered *via* KEGG analysis. Among them, eight diterpenoid biosynthesis genes were identified and highly expressed compared to other tissues. Finally, qRT-PCR verified 12 genes that were highest expressed in leaves were consistent with RNA-seq data (Kim et al., 2020). Shen et al. (2018) reported a draft *A. annua* genome sequence of 1.74 Gb that is assembled by Illumina and PacBio sequencing platform. They annotated 63, 226 protein-coding genes based on expression evidence. Gene expansion and contraction analysis revealed that 7,286 expanded and 3,950 contracted gene families in *A. annua*. Among the *A. annua* expanded gene families, TPS families expanded significantly and 122 TPS genes were identified. Subsequently, according to genomic and transcriptomic analyses, sesquiterpenoid synthesis-related genes were found and expressed in specific tissues. Previous studies were conducted to increase the yield of artemisinin by overexpressing the upstream or downstream enzymes, such as *FPS* and *HMGR*, but the artemisinin content did not increase significantly. Therefore, this study overexpressed the upstream, midstream and downstream enzymes [*HMGR (AA201470)*, *FPS*, and *DBR2*] simultaneously to examine the content of artemisinin, and the revealed that the transgenic plants produced more artemisinin. The present study provides a large number of candidate genes for further enhancement of artemisinin content. Xiong et al. (2021) revealed the *Taxus* genome with 10.23 Gb assembled into 12 chromosomes using Illumina HiSeq 2500, PacBio Sequel II, and Hi-C data. Based on genomic information and previous literature, 649 CYP450 genes were identified, of which CYP750 and CYP725 families in *Taxus* underwent significant expansion. Moreover, expression levels of four gene clusters on chromosome 9 where most CYP725A genes located on were significantly up-regulated by jasmonic acid induction. These results suggested that the gene cluster probably contains the majority of the paclitaxel synthesis genes that originated from the evolution of *T. chinensis*. PlantiSMASH analysis further indicated a gene cluster in group 9.2 that may be associated with terpenoid biosynthesis, including two TS genes, two T5αH genes, and two unknown CYP725As. Biochemical assays demonstrated that TS and T5αH were mainly responsible for catalyzing the first two steps of paclitaxel biosynthesis. Finally, 17 CYP725A genes which were closely related to known paclitaxel biosynthesis genes were identified through a gene-to-gene coregulation network. This article helps to accelerate paclitaxel biosynthesis and *Taxus* biotechnology applications.

### Flavonoids

Flavonoids are natural products with a C6-C3-C6 carbon skeleton structure, usually combined with sugars to form glycosides present in medicinal plants, which classified into flavonols, flavones, isoflavones, and anthocyanidins (Ciumarnean et al., 2020). Phenylalanine, the biosynthetic precursor of flavonoids, which is then converted to rutin through 10 enzymes, including phenylalanine deaminase (PAL), cinnamic acid-4-hydroxylase (C4H), 4-coumarate coenzyme A ligase (4CL), chalcone synthase (CHS), chalcone isomerase (CHI), flavanones-3′-hydroxylase (F3’H), flavanones-3′5’-hydroxylase (F3’5’H), flavonol synthase (FLS), glucose/witch hazel transferases (UGT/GT; Zhang L. et al., 2017). CHS is the first key enzyme in the flavonoid synthesis pathway and has been studied more in medicinal plants. In addition, O-methyltransferase (OMT) catalyzes the conversion of root-specific norbornin to other flavones, such as wogonin, isowogonin, and moslosooflavone (Liu W. et al., 2021). Zhao et al. (2019) reported the high-quality reference genome sequence of *Scutellaria baicalensis* at the chromosome level with 408.14 Mb (93%) assembled into 9 pseudochromosomes using Illumina, PacBio, and Hi-C technology. The article further elucidated the biosynthesis of norwogonin to wogonin catalyzed by 8-O-methyltransferase. Hence, 28,930 genes were annotated by bioinformatics tools based on *de novo* predictions, homology-based prediction, and RNA-seq data. On the basis of the genomic and transcriptome information, six genes encoding OMTs were found in the tandem repeat region unique to *S. baicalensis*. Moreover, enzyme activation and RNAi experiments confirmed that *SbPFOMT*s were involved in the synthesis of Wogonin, and the Wogonin synthesis pathway was resolved. It provides a basis for synthetic biology to obtain baicalein and a reference for genetic analysis of other Labiatae plants (Zhao et al., 2019). Wu et al. (2021) reported the first chromosome-scale reference genome of *Carthamus tinctorius* through combined PacBio platform and Hi-C mapping and predicted 33,343 protein-coding genes. Among them, seven CHS genes were identified based on the modified flavonoid biosynthetic pathway combined with the KEGG database and related literature. *CarCHS5* and *CarCHS6* which are unique to *C. tinctorius* were revealed by Collinearity analysis. These results provide evolutionary insights into the flavonoid biosynthesis in *C. tinctorius*. Qing et al. (2021) generated the *Hemerocallis citrina* genome of 3.77 Gb that was assembled by PacBio long reads and Hi-C data. Gene family expansion and contraction analysis revealed that 10,375 gene families in *H. citrina* underwent expansion, whereas a significant number of gene families (6707) underwent contraction. In addition, the expanded gene families were mainly enriched in flavonoids biosynthesis, which may have contributed to the content of rutin in *H. citrina*. Among them, 108 genes were identified by homology search. In addition, High-performance liquid chromatography/quadrupole time-of-flight (HPLC-Q-TOF) data revealed that rutin was mainly accumulated in flower buds, 20 candidate genes which mainly expressed in flower buds were identified in combination with transcriptome data. Zhang L. et al. (2017) reported a high-quality assembly of the 489.3 Mb genome of *Fagopyrum tataricum* at chromosome-scale, and contained 33, 366 protein-coding genes. In *F. tataricum* genome, nine rutin biosynthesis enzymes were identified, such as CHI (FtPinG0002790600) and F3’H (FtPinG0002353900). Although this study did not further validate the candidate genes, it provided some insights into the biosynthetic pathway of rutin.

### Alkaloids

Alkaloids are basic nitrogen-containing organic compounds with complex ring structures. It is mainly synthesized with precursors such as tryptophan, tyrosine, phenylalanine, lysine, and arginine. According to the structures, it can be classified into pyridines, indoles, terpenoids, isoquinolines, steroids, etc. (Liu et al., 2019). The previous report showed that CYP80 and CYP719 families catalyze many different reactions in the biosynthesis of BIAs, including hydroxylation, C-C, or C-O coupling, and formation of methylenedioxy bridges (Deng X. et al., 2018). Hu et al. (2021) presented the draft genome sequence of *Strobilanthes cusia*, constructed using PacBio long reads and Hi-C sequencing data. The draft genome assembly had a size of 913.74 Mb and contained 2,974 coding gene sequences, of which 2,975 DEGs were identified by transcriptome analysis and enriched in phenylpropanoid, flavonoid, and triterpenoid biosynthesis. Based on gene family expansion and contraction analysis, 60 gene families expanded in *S. cusia*, while 16 gene families contracted. In addition, these expanded gene families were mainly enriched in secondary metabolism pathway according to GO and KEGG analysis. On the basic of homology searching, 18 genes coding IA-related key enzymes were identified as DEGs in the *S. cusia* genome, such as *UGT*, *CYP450*, *ASA*, *TSB*, *BGL*, *CS*, and *EPSPS* genes. This study reveals the molecular basis of the accumulated indole alkaloids of *S. cusia*. Yang et al. (2021a) presented a reference chromosome level genome of *Areca catechu* by Illumina and PacBio data. The assembled genome was 2.51 Gb with a N50 scaffold size of 1.7 Mb and predicted 31,571 protein-coding genes. Among them, 904 expanded gene families were enriched in secondary metabolites pathways, including flavonoid, terpenoid, and isoquinoline alkaloid biosynthesis. Although this article was not mining deeper into functional genes related to secondary metabolite synthesis, it provides basic insights into areca alkaloid biosynthesis. Liu Y. et al. (2021) assembled a high-quality genome of *Coptis chinensis* at the chromosome level through integrating Nanopore sequencing, Illumina short reads, and Hi-C technology. Forty-one thousand four protein-coding genes were annotated. In addition, 1,083 gene families underwent expansion were revealed by gene evolution analysis. Among them, two new P450 families are found in early-diverging eudicots: CYP719 and CYP749. According to a comparison of these CYP719 genes, *Cch00017825* is clustered on chromosome 3 and expressed significantly in the *C. chinensis* rhizomes. Liu X. et al. (2017) reported the *Macleaya cordata* genome with 378 Mb using Illumina HiSeq 2000. Based on the previous report, 39 candidate genes related to SAN and CHE biosynthesis were identified. They performed liquid chromatography coupled with mass spectrometry (LC/MS) to detect the metabolites in *M. cordata* and obtained the SAN and CHE biosynthesis. Based on the reference genome of *M. cordata*, 39 genes were identified involved in SAN and CHE biosynthesis pathway. Additionally, SAN and CHE were not accumulated in the stem, 16 candidate genes were selected in combination with RNA-seq data. Finally, Metabolic engineering verified 14 candidate genes involved in catalytic reactions of SAN and CHE biosynthesis. This research could help medicinal plants produce more SAN and CHE. Rai et al. (2021) used PacBio long reads, Illumina short reads, Bionano optical mapping and Hi-C sequencing to assemble the *Ophiorrhiza pumila* genome. Based on metabolome annotation datasets, 273 nitrogen containing metabolites were annotated, most of them were indole alkaloids (IAs). According to a previous study and comparative genomic analysis, they found that monoterpene indole alkaloids (MIAs) biosynthesis are originated from strictosidine biosynthesis. In the late secoiridoid pathway, OG0014621 (*LAMT*) was found specifically expanded in *O. pumila*. Additionally, genes encoding MIA-related enzymes were replicated and obtained, such as *STR*, *SLS*, *7-DLH*, and *7-DLGT*. These suggested that gene clusters include several functional signature genes. The study presents a pangenome model of MIA biosynthesis that will help establish a sustainable supply of camptothecin. Kang et al. (2021) presented a high-quality genome sequence of *Camptotheca acuminata*, constructed using SMRT sequencing technology from PacBio, Illumina platform, and Hi-C techniques. In *C. acuminata*, gene expansion and contraction analysis indicated that 2,951 gene families expanded, while 1,733 gene families contracted. Previous research on *C. roseus* illustrated that CYP72A219 (SLS) and CYP72A224 (7-DLH) play a vital role in indole alkaloids biosynthesis in periwinkle. Hence, on the basic of homology searching and phylogenetic tress, two *SLS* genes (*CacGene13172*, *CacGene10833*) and two *7-DLH* genes (*CacGene13171*, *CacGene10832*) were identified in *C. acuminata* genome. This study reveals candidate genes that may play a role in the camptothecin biosynthesis in *C. acuminata*, providing a basis for future high-yield artificial biosynthesis.

### Quinone

Kang et al. (2020b) presented the chromosome-level genome of *Senna tora* with 526.4 Mb by PacBio long read sequencing and Illumina data and were assembled into 13 chromosomes using Hi-C data. Metabolite profile analyzed the content of 10 anthraquinone in different developmental stages of seeds and found that aurantio-obtusin was the major bioactive compounds in mature seeds. Furthermore, they predicted 45,268 protein-coding genes. Comparative genomics revealed that 2,874 gene families in *S. tora* underwent expansion, while 3,371 gene families underwent contraction. Interestingly, the expanded gene families were mainly enriched in phenylpropanoid, isoflavonoid, and terpene biosynthesis. Among the expanded gene families, 16 CHS-L genes were identified due to its rapidly expanded in in *S. tora*. At stage 4 when anthraquinones began to accumulate, two CHS-L genes (STO07G228250 and STO07G228220) presented high expression levels. Further phylogenetic tree analysis showed that STO07G228250 was more similar to HpPKS and ArOKS, octaketide synthases. Finally, according to ESI-MS spectrum and enzyme activity experiment, STO07G228250 was demonstrated to be involved in the first step of anthraquinone biosynthesis. The study provides a platform for medicinal plant *S. tora* with high bioactive molecular content.

### Phenylpropanoid

Yang et al. (2021b) constructed the assembly of 1.79 Gb genome sequence *of Arctium lappa* and obtained 32,771 protein-coding genes. Based on the genomic information, 616 positively selected candidate genes were discovered in *A. lappa*. Transcriptome analysis revealed that genes related to lignan biosynthesis in five different stages of *A. annua* (4CL), dirigent protein (DIR), and hydroxycinnamoyl transferase (HCT) were highly related to arctiin biosynthesis.

## Pharmacodynamic Genes Mining for the Chronic Complex Diseases

Herbs have been demonstrated in studies to help with the treatment of rheumatism, diabetes, cancer, Alzheimer’s disease, and cardiovascular disease (Li X. et al., 2019). Take rheumatism as an example, they treat rheumatism by the following effects: dispelling wind, eliminating the body moisture, removing coldness, clearing heart, dredging the channel, expectorant and diffusing impediment, benefiting Qi and nourishing the blood, and invigorating the kidney and strengthening the spleen.

### Rheumatism

*Callerya speciosa* made contributions to treat rheumatism *via* dredging the channel effects, due to its main medicinal ingredients-isoflavonoids, such as maackiain and formononetin. Yao et al. (2021) used NGS technology to sequence the transcriptome of *C. speciosa* and identified 4,337 DEGs during the tuberous root development. Among them, 15 genes related to isoflavonoids biosynthesis were found. These results indicated that these genes may be promoted the accumulation of isoflavonoids in the tuberous toot. In addition, qRT-PCR validated the expression pattern of candidate DEGs were consistent with the RNA-seq data. The study provides new insights into the potential mechanisms of isoflavonoid biosynthesis in *C. speciosa*. Flavonoid, a bioactive compound derived from *Fritillaria hupehensis*, has often been used as an expectorant to treat rheumatic diseases. Guo K. et al. (2021) performed SMART analysis from the PacBio platform to sequence the full-length cDNA of *F. hupehensis*. Thirty-four flavonoid biosynthesis unigenes were found using the KEGG pathway, and divided into five branches by blast against model plants. The study provides a valued resource for herb breeding and bioactive compounds for pharmacological application. *Asarum sieboldii* has abundant medicinal properties, such as anti-inflammatory, antiallergic, and removing coldness. Asarinin and aristolochic acid are bioactive compounds that originated from *A. sieboldii*. Chen C. et al. (2021) used full-length transcriptome analysis to uncover genes involved in asarinin and aristolochic acid biosynthesis. The result found 63, 023 transcriptional sequences, of which 41 asarinin biosynthesis candidate genes and 56 aristolochic acid biosynthesis candidate genes were identified, including *AsCOMT*, *AsEPI*, *AsCYP81Q2*, *AsCYP81Q4*, *AsCYP81Q7*, *AsCYP81Q29*, *AsTYR*, *AsTYDC*, *AsNCS*, *AsNOMT*, *AsCNMT*, and *AsCYP80B1*. Finally, qRT-PCR data verified 4 genes were significantly expressed in the root, including *AsCCR*, *AsPAL*, *AsCOMT*, and *AsCYP81Q*. The study will provide a good basis for the production of a valuable, low toxicity active ingredient. *Gynostemma pentaphyllum* has the pharmacological effect of eliminating the body moisture and clearing the heart. Gypenosides, triterpene saponins, are the main active compounds in *G. pentaphyllum*. Liang et al. (2019) obtained 140,157 unigenes using PacBio standard analysis pipeline and Illumina data. Among them, 404 gypenoside biosynthetic genes were detected and annotated. GpOSC1, GpCYP89, and GpUGT35 were demonstrated the leading candidate genes for gypenoside biosynthesis by qRT-PCR technology. These findings will lay a new foundation for gypenosides biosynthesis. *Akebia trifoliata* possesses the properties of strengthening the spleen and relieving pain. Bioactive compounds contribute medicinal effects to *A. trifoliata*, including triterpenoid saponins, triterpenes, and flavonoids. Huang H. et al. (2021) presented the chromosome level genome sequence of *A. trifoliata* using Illumina HiSeq X-Ten sequencing technology, SMRT platform from PacBio and Hi-C technique. The genome assembly had a size of 682.14 Mb and predicted 25,598 protein-coding genes. Two hundred forty-six expanded and 473 contracted gene families in *A. trifoliata* were discovered, according to the phylogenetic tree. Interestingly, the expanded gene families were mainly enriched in terpenoid biosynthesis *via* KEGG enrichment analysis. Among the expanded gene families, 24 *Atrβ-AS* genes, 12 UDP-glucoronosyl, three UDP-glucosyltransferase and seven cytochromes P450 gene families were involved in sesquiterpenoid and triterpenoid biosynthesis pathways. In addition, three UDP-glucosyltransferase, 14 cytochrome P450, and two TPS gene families were constructed in the *A. trifoliata* genome. The findings suggested that these gene families were quickly changing to synthesis varies of triterpenes in *A. trifoliata*. The study provides a useful genetic resource for pharmacological applications of *A. trifoliata*. *Trillium govanianum* contributes to the treatment of rheumatism disease, due to its ability to benefit Qi and nourish the blood. Diosgenin, as steroidal saponins, is considered to be the main bioactive component of *T. govanianum*. Singh et al. (2017) performed spatial transcriptome analysis of the leaf, fruit, stem, and rhizome tissues of *T. govanianum* and obtained 69,174 transcripts. As a result, 108 CYP genes and 58 UGTs were identified, of which 87 CYP genes and 49 UGTs were differentially expressed in four tissues. Based on KEGG classification, genes involved in steroidal saponin biosynthesis were divided into three pathways: terpenoid backbone (16 genes), sesquiterpenoid and triterpenoid (two genes), and steroid biosynthesis (14 genes). In addition, qRT-PCR was employed to confirm the expression pattern of 29 genes, which was consistent with the RNA-seq results. Thus, compared with transcriptome sequencing, *de novo* genome assembly of herbs mines more functional genes involved in the biosynthesis pathways of active ingredients, which is more beneficial to the development of herb molecular breeding.

### Diabetes

For many years in India, *Gymnema sylvestre*, a well-known and valuable medicinal plant, has been used to treat diabetes (Hossain et al., 2016; Tiwari et al., 2017; Pham et al., 2018). Ayachit et al. (2019) performed RNA-seq data to uncover terpenoid biosynthesis genes and identified 111 transcripts involved in the terpenoid biosynthetic pathway, such as mono-terpenes, di-terpenes, tri-terpenes, and ubiquinones. Finally, qRT-PCR verified six transcripts involved in the MEP pathway were a positive correlation to terpenoid biosynthesis. This study provides insights for future functional genomics studies of *G. sylvestre*. *Eriobotrya japonica*, a traditional medicine, is beneficial in the treatment of diabetes, due to its variety of active compounds, such as flavonoids and terpenoids (Mogole et al., 2020). Wang (2021) constructed a draft genome of *E. japonica* to discover medicinal bioactive compounds using HiSeq 4,000 sequencing platform, PacBio long-read sequencing technology, and Hi-C data and obtained 45,492 protein-coding genes. According to gene family expansion and contraction analysis, 483 gene families in *E. japonica* expanded significantly and were mainly enriched for metabolic pathways in combination with KEGG analysis. Metabolite profiles showed that phenolic acids, flavonoids, and terpenoids were detected abundantly in the *E. japonica*. Based on genomic information, 71 flavonoid biosynthesis genes were annotated, of which 3 genes encoding key enzyme were identified in the quercetin biosynthesis pathway. In addition, 286 predicted protein-coding genes in phenylpropanoid biosynthesis were identified, only five genes underwent in the expansion family. According to KEGG analysis, 92, 32, 56, and 37 candidate genes were identified involved in terpenoid backbones, monoterpenoids, diterpenoids, and sesquiterpenoid-triterpenoids biosynthesis pathways, respectively. The study provides a valuable introduction for further molecular pharmacological studies of *E. japonica*. Thousands of years ago, *Pueraria thomsonii* was used to treat diabetes in the East. Puerarin as the bioactive isoflavones is mainly accumulated in the root of *P. thomsoni* and has antioxidant and anti-inflammatory properties (Chen et al., 2018; Yang et al., 2019; Luo et al., 2021). He et al. (2019) performed PacBio and Illumina sequencing technology to sequence the *P. thomsoni* transcriptome and acquired 44,339 transcripts. They discovered 9,225 differentially expressed transcripts (DETs). Among them, 32 genes might be involved in isoflavone production, of which the expression profile of eight genes were confirmed by qRT-PCR which consistent with RNA-Seq data. *Glycyrrhiza uralensis*, an important medicinal plant of the genus *Glycyrrhiza*, has been used as TCM. Flavonoids and Glycyrrhizin originated from liquorice possess antioxidative, antidiabetic, and anti-inflammatory effects (Lee et al., 2010; Feng et al., 2013; Lin et al., 2022). Mochida et al. (2017) reported a draft genome of *Glycyrrhiza uralensis*, based on Illumina short reads and PacBio long reads. The assembled genome is 379 Mb with scaffold N50 of 109 kb, encoding 34,445 predicted genes. On the basis of genomic information, *CYP93C*, *HI4OMT*, and *7-IOMT* some of which are involved in isoflavonoid biosynthesis were observed and generated a cluster. Based on homolog searching and functional annotation, P450 and UDP-dependent glycosyltransferase (UGT) families involved in triterpenoid saponin biosynthesis were predicted and the expression of *bAS*, *CYP88D6* and *CYP72A154* were consistent with the glycyrrhizin yield of the *G. uralensis* samples. In addition, RNA-seq data revealed that Glyur002597s00038051 and Glyur002597s00038050 which closely homologous to CYP72A9 and UGT73B1 in *Arabidopsis thaliana* were high correlation of expression patterns. Hence, P450 and UGT genes might be involved in triterpene saponin biosynthesis in *G. uralensis*. These findings help researchers use genomic resources combined with biosynthetic approaches to create a library of rare natural or novel bioactive compounds to facilitate drug discovery. *Sophora flavescens* are important traditional medicinal plants with pharmacological properties effective in the treatment of inflammatory disorders including diabetes complications (Guo et al., 2021b). Alkaloids and flavonoids are the major bioactive compounds in root tissues. Wei et al. (2021) performed transcriptome analysis of the periderm, phloem, and xylem tissues of *S. flavescens* and obtained 58,327 unigenes. High-performance liquid chromatography (HPLC) detected metabolite contents in the root tissues and the results showed that alkaloids contents were highest in the phloem, while flavonoids contents were highest in the xylem. Fifty-two and one hundred thirty-seven CYP transcripts involved in alkaloid biosynthesis were identified and expressed highest in the xylem. Additionally, 37 transcripts were found in flavonoid biosynthesis and expressed highest in the xylem. Correlation analysis found LYSA, AO, PMT transcripts were markedly and positively correlated with alkaloids contents and 4CL, 2’OH, CHI5, CHRI transcripts were markedly and positively correlated with flavonoids contents. These results provide a basis for the molecular breeding of *S. flavescens*.

### Alzheimer’s Disease

Curcumin, the bioactive compound of *Curcuma longa*, may prevent or reverse Alzheimer’s disease due to its anti-inflammatory and antioxidant activity (Kim et al., 2022). Chakraborty et al. (2021) assembled the draft genome sequence of *C. longa* using Oxford Nanopore long reads and obtained 50,401 coding gene sequences. Among 10 enzymes involved in the curcuminoid biosynthesis pathway, gene family evolution analysis revealed that two gene families (*HCT* and *OMT* genes) appear to be undergoing contraction, while a significant number of gene families (8) appear to be expanding in *C. longa*. The result suggests that genes related to the curcumin biosynthesis pathway have evolved, providing a genetic basis for its pharmacological properties. *Corydalis yanhusuo* have been used to relieve neuropathic pain, due to its bioactive compounds-tetrahydropalmatine, a member of BIAs. Xu D. et al. (2021) performed SMRT sequencing from the PacBio platform to sequence the cDNA library from leaves and tubers of *C. yanhusuo*. Based on the tblastn results, 101 unigenes involved in BIA biosynthetic pathway were founded, of which 36 unigenes were identified as DEGs *via* expression analysis, owing to the majority of them expressed at a higher degree in tubers which consistent with the metabolome data. In addition, phylogenetic analysis showed that 10 OMT unigenes involved in the final step for tetrahydropalmatine synthesis were identified. This study provides the basis for the subsequent molecular cloning and activity validation of the enzyme. Diosgenin is an anti-inflammatory and antioxidant compound found in the rhizome of *Dioscorea zingiberensis*. Cheng J. et al. (2021) used Illumina HiSeq and PacBio SMART technologies to sequence the *D. zingiberensis* genome and annotated 26,022 protein-coding genes. Based on previous study and genomic information, they found *DzinCYP90G6* and *DzinCYP94D144*, two P450 genes, were related to diosgenin biosynthesis using blastp queries. Finally, metabolic engineering verified that coexpression of *DzinCYP90G6* and *DzinCYP94D144* in yeast was able to produce diosgenin. This study helps to decode the evolutionary trajectory of the biosynthetic pathway of diosgenin, but also provides insights into the enhancement of diosgenin production through biochemical synthesis. Chlorogenic acid (CGA) accumulates in the leaves and bark of *Eucommia ulmoides*, and it can reduce the concentration of glucose in the blood after the meal and lower blood pressure. Li et al. (2020) used PacBio Sequel platform, Illumina NovaSeq platform, and Hi-C data to assemble a high-quality *E. ulmoides* genome. Twenty-six thousand one protein-coding genes were predicted. Based on homologous gene comparison in the *E. ulmoides* genome, 23 candidate genes encoding six key enzymes involved in the CGA biosynthesis pathway were identified, including PAL, 4CL, C4H, C3′H, HCT, and HQT genes. In addition, gene expression profile analysis showed that the expression levels of these genes were higher than other genes. This work will accelerate the understanding of the molecular mechanisms of biosynthesis of other valuable secondary metabolites, such as rutin and quercetin. Dihydroquercetin (DHQ) is a pharmacologically active, which can be converted to dihydromyricetin (DHM). Yu Z. et al. (2021) used HPLC to detect the DHM content in *Ampelopsis grossedentata* from different geographical locations and divided into two groups: B group (low DHM) and D group (high DHM). Then, Illumina HiSeq 2000 sequencing platform was performed to sequence the transcriptome of *A. grossedentata* from B and D groups and annotated 57,016 unigenes in D group using seven public protein databases. The differentially expressed gene analysis revealed 926 DEGs in B vs. D, of which 446 up-regulated genes and 480 down-regulated genes. DEGs of 10 structural enzyme genes associated with flavonoids biosynthesis were identified, including *PALs*, *CLs*, *CHSs*, *F3’H*, *F3’5’H*, *ANS*. In addition, qRT-PCR verified the expression level of selected genes which was matched the transcriptome data, including *CHSs*, *F3’H*, and *F3’5’H*. This work will stimulate further genetic research on *A. grossedentata* and may ultimately lead to genetic improvements in the plant’s DHQ content.

### Cancers

**Figure 3:**
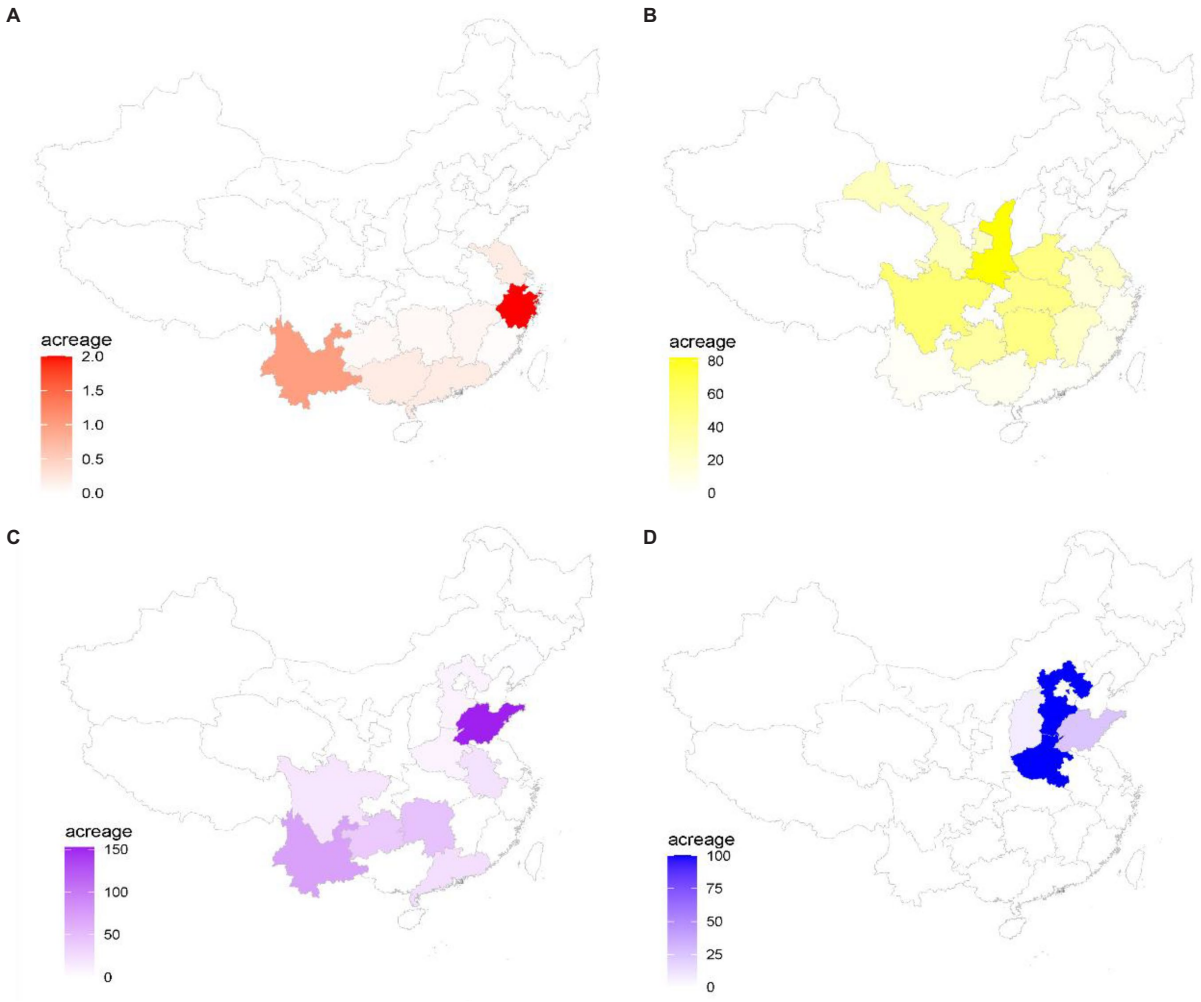
Industry distribution and covered areas of four representative species in China. **(A)** *Dendrobium officinale*; **(B)** *Eucommia ulmoides*; **(C)** *Zingiber officinale*; and **(D)** *Dioscorea zingiberensis*. A color scale bar is shown the acreage size of species in each province of China.

*Catharanthus roseus* accumulates vinblastine and vincristine, which have long been known to be an anti-cancer drug to cure glioma disease (Skubnik et al., 2021). Verma et al. (2014) performed Illumina platform to sequence the *C. roseus* transcriptome from leaf, flower, and root and obtained 59,220 unique transcripts. Next, using the CathaCyc database analysis, 30 well-known genes involved in TIA biosynthetic pathways were identified and they are conserved in the sequenced reference genome of tomato, potato, and Arabidopsis at the protein level. Based on the RNA-seq data, most of TIA biosynthesis genes were up-regulated in leaf and root tissues, while Cr_TC35206 and Cr_TC35622 were highly down-regulated in roots, Cr_TC04217 were highly down-regulated in leaves. Finally, they performed qRT-PCR to validate 10 TIA biosynthetic pathway genes and their expression pattern consistent with RNA-seq data (see Figure 2). This research adds to our knowledge of the regulatory systems that control alkaloid production. *Andrographis paniculata* produces a large number of diterpenoid lactones with antitumor and immunomodulatory effects which are usually used to treat esophageal cancer, including andrographolide and neoandrographolide (Li L. et al., 2017, 2019; Sun et al., 2019; Yue et al., 2019). Sun et al. (2019) assembled *A. paniculata* genome sequence of 269 Mb at chromosome-scale using Illumina short reads, PacBio long reads, and Hi-C data and predicted 25,428 protein-coding genes. Based on the phylogenetic tree, 1,290 expanded and 5,383 contracted gene families in *A. paniculate* were discovered. Interestingly, the expand gene families were mainly enriched in the secondary metabolism pathway, such as TPS and CYP gene families, which may have contributed to the synthesis of andrographolide and neoandrographolide. Previous reports suggested that CYPs and 2OGDs may be involved in turing the diterpene backbone into diterpenes. Therefore, 278 CYP genes and 112 putative 2OGDs in the *A. paniculata* genome. Moreover, diterpene lactones need to be glycosylated to become neandrographolide, and 120 putative UGT genes were identified. Transcriptome data revealed that 18 CYP71and 6 CYP76 family members, 17 2OGDs, and 29 ApUGTs were significantly elevated after MeJA treatment. Finally, UGT73AU1 was confirmed that it converted the andrograpanin to neandrographolide *via* enzymatic assays. The study provides further understanding of the production of bioactive diterpene lactone components. The antitumor and anticancer medicinal properties of *Lantana camara* are attributed to its multiple bioactive substances, such as triterpenod which can be used to treat papilloma (Sharma et al., 2007). Shah et al. (2020) performed Illumina sequencing platform to analyze *L. camara* transcriptome in leaf and root and found 72,877 and 513,985 unigenes from leaf and root tissues, respectively. Moreover, 229 and 943 genes involved in the phenylpropanoid biosynthesis in leaf and root tissues, respectively were identified by pathway analysis. Twenty thousand forty-four DEGs were identified. Among them, 11,496 genes were up-regulated and 8,548 genes were down-regulated. The transcriptome analysis provides the basis for further molecular studies of *L. camara*. *Salvia officinalis* is used in medication for treating liver cancer, leukemia, colon cancer through inhibiting cell proliferation and apoptosis (Jantová et al., 2014; Pedro et al., 2016; Jiang et al., 2017). Ali et al. (2017) used Illumina HiSeq 2000 to sequence the transcriptome of *S. officinalis* leaves and assembled 48,671 unigenes. Among them, 65 unigenes involved in terpene synthase were identified, according to the TPS sequence in the reference database. Furthermore, 11 DEGs were selected to be validated by qRT-PCR. The results showed that eight candidate genes were most highly expressed in young leaves, except for *SoGGPS*, *SoLINS*, and *SoHUMS* genes were most highly expressed in stems. qRT-PCR data are well in agreement with GC–MS analysis data, major terpene groups present in young leaves. In addition, five terpene synthase genes (*SoNEOD*, *SoCINS*, *SoSABS*, *SoLINS*, and *SoTPS6*) were selected to overexpress in tobacco, respectively. The results indicate that more terpenoids were produced in transgenic tobacco. 2-methoxy-1,4-naphthoquinone (MNQ) is an active component of *Impatiens balsamina* with anticancer pharmacological properties. Shikimate and 1,4-dihydroxy-2-naphthoate (DHNA) pathways are responsible for the synthesis of MNQ, which lawsone is first synthesized by oxidative decarboxylation of DHNA and then possibly catalyzed by S-adenosylmethionine-dependent O-methyltransferase (SAM-dependent O-MT), NADH-quinone oxidoreductase, and UDP glycosyltransferases. Foong et al. (2020) reported the transcriptome sequencing of *I. balsamina* through Illumina HiSeq4000 paired-end sequencing technology and obtained 50,786 unigenes, of which 27, 104, 82, and 122 unigenes related to DHNA pathway, SAM-dependent O-MT activity, NADH-quinone oxidoreductase, and UDP glycosyltransferases, respectively. Among them, five unigenes related to DHNA pathway were highest expressed in early-stage capsule, gene expression of six unigenes were substantially and positively connected with MNQ content, 3 NADH-quinone oxidoreductase and 5 UDP glycosyltransferases were connected with lawsone content significantly. In addition, HPLC analysis indicated that lawsone was highest expressed in mature leaves, whereas MNQ was expressed only in capsules pericarps. Finally, qRT-PCR verified 20 candidate genes were consistent with transcriptome data. *Dendrobium officinale* is a widely used medicinal plant that creates a variety of bioactive compounds which resist cancer by inhibiting cell proliferation (Luo et al., 2019; Tao et al., 2021). Niu et al. (2021) reported its 1.23 Gb genome, encoding 27,631 predicted genes using PacBio long-reads, Illumina short-reads, and Hi-C data. Additionally, gene family evolution analysis found that 820 gene families expanded in *D. officinale*, while 975 gene families contracted. Interestingly, the expanded gene families were mainly enriched in polysaccharides, alkaloids and flavonoids biosynthesis according to KEGG enrichment analysis. Based on homolog searching and functional annotation, 268, 98, and 52 genes related to three main bioactive ingredients biosynthesis were identified, including polysaccharides, alkaloids, and flavonoids biosynthesis, respectively. Differential expression profiling revealed 1,677 DEGs, of which 51 DEGs related to polysaccharides, alkaloids, and flavonoids biosynthesis were identified. Additionally, 218 *CYP450* genes were found in the *D. officinale* genome according to homologous search from *A. thaliana*, of which 29 DEGs were identified using comparative transcriptome analysis and most of them are CYP71 family members which were up-regulated. The results suggested that CYP71 groups make a great contribution in regulating the synthesis pathway of active ingredients. Therefore, the high-quality reference genome reported in this study can contribute to the functional genomics study and molecular breeding of *D. officinale*. Chen et al. (2012) reported a reference genome assembly of *Ganoderma lucidum* using Illumina next-generation sequencing and predicted 16,113 genes. Terpenoids and polysaccharides are the main active components of *G. lucidum*. One hundred ninety-seven CYP functional genes were identified, of which 78 CYP genes expression levels were consistent with terpenoids content in *G. lucidum*. According to previous literatures, 15 CYP512 and one CYP5144 genes were discovered in the *G. lucidum* genome, which may be related to triterpenoid biosynthesis. In addition, two polysaccharide genes, two LZ-8 genes, 12 TPS genes were identified. This study will pave the way for the role of *G. lucidum* in pharmacological and industrial applications (see Figure 3). *Zingiber officinale*, a gingerol-producing medicinal plant, possesses anti-cancer properties and treats breast cancer by inhibiting apoptosis (Huang P. et al., 2021). Li H. et al. (2021) reported chromosome-level reference genome assembly of *Z. officinale* using PacBio long reads, Illumina short reads, and Hi-C reads. Based on the assembly *Z. officinale* genome, they investigated the gene family expansion and contraction and revealed that 1,098 gene families underwent expansion, while 20 gene families underwent contraction. In addition, two expanded gene families (*PKS* and *AOR*) were mainly enriched in the biosynthesis of secondary metabolites. UHPLC–MS/MS detected rhizomes of *Z. officinale* at different stages and revealed that the concentration of active compounds were the lowest in R1, whereas were the highest in R5, such as gingerol analogs-6-gingerol and tetrahydrocurcumin. According to the metabolomic data and previous study, 12 gene families involved in the gingerol biosynthesis pathway were identified, and the expression level of 10 gene families were compatible with the concentration of gingerol. Additionally, *C3OMT2*, *C3OMT3*, and *C3OMT13* formed a unique clade in the *Z. officinale* genome which suggested that C3OMTs were likely related to feruloyl-CoA biosynthesis.

### Cardiovascular Disease

The tubers of *Ophiopogon japonicus* are accumulated in many active substances which contribute significantly to the treatment of cardiovascular diseases through their antioxidant, anti-inflammatory, and cardioprotective pharmacological properties, such as flavonoids, saponins, and polysaccharides (He et al., 2016; Wu et al., 2019; Fan S. et al., 2020). Liu H. et al. (2017) employed the Illumina platform to sequence the transcriptome of different ages tubers (Y1, Y2, Y3) from *O. japonicus* and generated 96,738 unigenes with *de novo* assembly. According to searching against the five databases, 77,409 unigenes were annotated among the 96,738 unigenes. Based on the result of gene annotation, 245, 135, and 236 unigenes encoding key enzymes in flavonoid, saponin, polysaccharide biosynthesis were identified, respectively. Most genes related to polysaccharide biosynthesis have the highest expression in Y2, while genes involved in flavonoid and saponin biosynthesis have the highest expression in Y1. qRT-PCR verified the expression level of 17 unigenes were consistent with RNA-Seq data, which were selected from flavonoid, saponin, polysaccharide biosynthesis, respectively. This study can accelerate the understanding of the physiological process of active ingredient synthesis at the molecular level and promote the development of natural drugs. Chrysanthemums have significant protective functions for cardiovascular (Song et al., 2018). Song et al. (2018) performed Oxford Nanopore long reads and Illumina short reads to assemble the genome of *Chrysanthemum nankingense*. In *C. nankingense*, gene evolution analysis revealed that 1965 gene families underwent expansion, while a significant number of gene families (1777) underwent contraction. Surprisingly, the expanded gene families were mainly enriched in terpene synthase activity. Among them, 219 TS genes and 708 CYP genes were identified, including seven SQSs, 158 TPSs, and 54 TCCs. Due to, the genes that make up the terpene synthesis pathway are organized into gene clusters, TPS-a/CYP99 and TPS-g/CYP79/CYP76, a new combination, were found in the *C. nankingense* genome. Further analysis revealed CHR00048430 and CHR00048432 genes from the TPS-a/CYP99 cluster were mainly expressed in roots, whereas CHR00011805 and CHR0001181 from TPS-g/CYP79/CYP76 cluster were mainly expressed in flowers. Additionally, two key flavonoid biosynthesis genes (*CHS* and *CHI*) from expansion families were significantly highly expressed in flowers. The study gives genomic data to help researchers better understand the evolutionary history of chrysanthemums. *Rehmannia glutinosa* possesses a significant effect on treating cardiovascular disease accumulation of iridoids in root tissues is thought to be responsible for its health advantages (Ma L. et al., 2021). Ma L. et al. (2021) reported a chromosome level of 2.49 Gb genome sequence of *R. glutinosa* and were assembled into 14 chromosomes using Illumina NovaSeq reads, Oxford Nanopore technology, and Hi-C data. Most iridoids exist mainly as glycosides, due to glycosylation being the final step in terpene biosynthesis. In addition, gene family evolution analysis revealed that 6,237 gene families in *R. glutinosa* underwent expansion, whereas 848 gene families underwent contraction. Eighty-seven TPS and 333 UGT genes were discovered in the expanded gene families, with the majority of them being significantly expressed in roots. The genomic resources provided by this study are essential for the molecular breeding of medicinal plants. Previous research reported that flavonoids derived from *Ziziphora bungeana* possess the ability to treat cardiovascular disease (Li et al., 2018). He et al. (2020) performed Illumina technology to sequence the transcriptome of *Z. bungeana* from different tissues and assembled 397,182 unigenes. Firstly, LC/MS detected 12 flavonoid components and linarin was one of the main bioactive compounds which highly accumulated in the inflorescence. Secondly, based on the transcriptome data, 18 candidate genes were identified from assembled unigenes that encode four key enzymes, including PAL, C4H, 4CL, and FNSII. Finally, qRT-PCR data revealed that *ZbPAL3*, *Zb4CL3*, *ZbCHS1*, *ZbFNSII*, and *ZbANS* had the highest expression levels in inflorescence, which suggested that they were likely related to the lignin biosynthesis.

## Conclusion and Outlook

Research on medicinal plants has focused on the genetic mechanism of active ingredients synthesis and the subsequent pharmacological effects on diseases treatment. Thus, when researchers collect samples, they need to pay attention to the geographical distribution, different tissues and different developmental stages of the herb, due to these aspects play an important role in the efficacy of the pharmacological components (Duan et al., 2014; Zhang X. et al., 2017). The characteristics summary of the CHM is shown in Table 2. NGS had been widely applied in transcriptome sequencing of medicinal plants to get the expression profile and mined the genes related to bioactive compounds before the advent of TGS. However, due to the short read length, it was not discovering more candidate genes involved in the synthesis of active ingredients. With the aid of matured sequencing technologies, TGS is used to sequence the full-length transcriptome of medicinal plants, making a name for itself in herbal medicine research. *Salvia miltiorrhiza*, a well-known herbal medicine, is commonly used to treat diabetes. It has various biological functions, such as anti-oxidative stress and anti-inflammatory (Jia et al., 2019; Guo Y. et al., 2021; Yin et al., 2021). For instance, Xu et al. (2015) used SMRT *via* PacBio long reads to sequence RNA mixes from three root tissues of *S. miltiorrhiza* and obtained high-quality full-length transcriptome sequences. Furthermore, using Illumina short reads to quantify gene expression in three different tissues and obtained DEGs which elucidated the molecular mechanism of salvinone accumulation in the periderm. What’s more, alternative splicing analysis on the full-length transcripts revealed that many terpenoids and isoprene biosynthesis genes underwent alternative splicing. Based on the above findings, the results suggest that these genes may regulate the diterpene metabolic pathway. Nevertheless, comprehensive analysis of multi-omics data is required for mining pharmacodynamic genes more accurately, such as genomics, transcriptomics, metabolomics, and so on (Ma et al., 2020). Ma Y. et al. (2021) revealed the *S. miltiorrhiza* genome with 622 Mb using Illumina Hiseq2000 and PacBio RS platform and obtained 33,760 protein-coding genes. CYP450 genes were discovered to be present as a gene cluster after a thorough study of genomic data. Based on this, a CYP71D subfamily, which is significantly expanded in the *S. miltiorrhiza* genome, was identified using gene expansion and contraction analysis, and four candidate genes were targeted by co-expression analysis for enzyme activity and RNAi studies. The results showed that three of these genes play important roles in the tanshinone biosynthetic pathway, two of which can catalyze the generation of tanshinone characteristic furan rings and one is associated with the hydroxylation process at the C20 position of tanshinones. Based on previous literature, the researchers used the genome-assembly and the downstream bioinformatics analysis to uncover key enzymes genes involved in the biosynthesis pathway of active ingredient. Furthermore, the validation methods for functional genes are shown in Table 1.

**Table 2:** Characteristics summary of the CHMs.

Name	Chinese name	Bioactive compounds	Representative medicinal tissue	Medical applications	Industrialized	References
*Aconitum carmichaelii*	乌头	Atisine, napelline, and aconitine	Roots	Treat pain, rheumatics, heart failure, diarrhea, beriberi, and edema	Yes	Mao et al., 2021
*Akebia trifoliata*	白木通	Oeanolic acid	Stems	Anti-inflammatory, antimicrobial, antioxidative, and anticancer	Yes	Huang H. et al., 2021
*Ampelopsis grossedentata*	显齿蛇葡萄	Dihydroquercetin and dihydromyricetin	Immature leaves	Antioxidants, anti-inflammatory, anti-microbial, anti-cancer, anti-Alzheimer, and anti-toxoplasmosis	Yes	Yu Z. et al., 2021
*Andrographis paniculata*	穿心莲	Neoandrographolide	Stems and leaves	Treat infectious, lung diseases, diabetes, and cancer	Yes	Li L. et al., 2017, 2019; Sun et al., 2019; Yue et al., 2019
*Angelica sinensis*	当归	Ferulicacid and soluble sugar	Roots	Treat irregular menstruation	Yes	Gao et al., 2021
*Arctium lappa*	牛蒡	Lignans	Fruits and roots	Treat common cold, measles, rubella, and mumps	Yes	Yang et al., 2021b
*Areca catechu*	槟榔	Arecoline	Seeds and nut	Treat nasal ulcers, Alzheimer’s disease and obesity	Yes	Yang et al., 2021a
*Arisaema heterophyllum*	天南星	Terpineol, linoleic acid, schaftoside, and isoschaftoside	Tuber	Anti-tumor, antibacterial, anticonvulsant, analgesic, and anti-inflammatory	Yes	Wang C. et al., 2018
*Artemisia annua*	青蒿	Artemisinin	Whole plant	Treat uncomplicated malaria, tuberculosis, diabetes, and cancer	Yes	Shen et al., 2018
*Artemisia argyi*	艾蒿	Lupenone, β-amyrin, and simiarenol	Leaves	Antibacterial, anti-malarial, cough relief, anti-cancer, anti-diabetes, and anti-diarrhea	Yes	Cui et al., 2021
*Asarum heterotropoides*	库叶细辛	Aristolochic acid	Roots and rhizomes	Treat wind chill, headache, and cough	No	Wang X. et al., 2018
*Asarum sieboldii*	细辛	Asarinin and aristolochic acid	Whole plant	Treat cough, ache, inflammation, and cancer	Yes	Chen C. et al., 2021
*Astragalus membranaceus*	黄芪	Astragalosides, calycosin, and calycosin-7-O-β-D-glucoside	Roots	Treat cardiovascular disease, type 2 diabetes, nephritis, and cancers	Yes	Li J. et al., 2017
*Berberis koreana*	掌刺小檗	Berberine	Stems and roots	Antibacterial, anti-convulsion, sedative, anti-cholinergic, cholagogic, hepatoprotective, and anticancer	Yes	Roy et al., 2021
*Callerya speciosa*	鸡血藤	Maackiain and formononetin	Tuberous roots	Enhance immunity, hepatoprotection, arresting cough, and expectorant and anti-asthmatic	Yes	Yao et al., 2021
*Camptotheca acuminata*	喜树	Camptothecin	Leaves and fruits	Treat malignant tumors	Yes	Kang et al., 2021
*Carthamus tinctorius*	红花	Hydroxysafflor yellow A and linoleic acid	Flowers and seeds	Treat gynecological, cerebrovascular, and cardiovascular	Yes	Wu et al., 2021
*Cassia obtusifolia*	钝叶决明	Aurantio-obtusin	Seeds	Treat dizziness, headache, and diabetes	Yes	Deng Y. et al., 2018
*Catharanthus roseus*	长春花	Vinblastine and vincristine	Whole plant	Treat diabetes, cardio-vascular disorders, and anti-cancer	Yes	Verma et al., 2014
*Chrysanthemum nankingense*	菊花脑	Linalool, β-farnesene and lemon oil	Stems and leaves	Antioxidative, cardiovascular protective, vasorelaxant, and antivirus	Yes	Song et al., 2018
*Coptis chinensis*	黄连	Berberine, coptisine, and worenine	Rhizomes	Treat cardiovascular, diabetes, cancer, and the nervous system diseases	Yes	Chen D. et al., 2021; Liu Y. et al., 2021
*Cordyceps guangdongensis*	冬虫夏草	Cordycepic acid and adenosine	Whole plant	Cure chronic renal failure	Yes	Zhang et al., 2018; Chen Y. et al., 2019
*Corydalis yanhusuo*	延胡索	Sanguinarine and noscapine	Tubers	Treat cardiovascular diseases, cancer, enhance energy, and provide analgesia	Yes	Xu D. et al., 2021
*Crocus sativus*	番红花	Apocarotenoid	Stigmas	Alleviate cramps, depression, anxiety, cardiovascular diseases, nervous disorders, and cancer	Yes	Yue et al., 2020
*Curcuma longa*	姜黄	Curcumin	Roots	Anti-inflammatory, antioxidant, and anti-cancer activities	Yes	Kim et al., 2022
*Dendrobium officinale*	铁皮石斛	Cyanidin, dihydroquercetin, fomononetin, biochanin A, and chorismate	Stems	Benefit the stomach, resist cancer, and enhance the body’s immunity	Yes	Luo et al., 2019; Niu et al., 2021; Tao et al., 2021
*Entada phaseoloides*	榼藤	Palmitic acid and entada saponin	Stems	Dispel wind and body moisture, exhibit anti-inflammatory activity	No	Liao et al., 2020
*Eriobotrya japonica*	枇杷	Citric acid, nerolidol, and crategolic acid	Leaves	Treat inflammation, diabetes, cancer, bacterial infection, aging, pain, and allergy	Yes	Mogole et al., 2020; Wang, 2021
*Eucommia ulmoides*	杜仲	Chlorogenic acid	Leaves and bark	Reduce blood pressure and antibacterial, antiviral, antitumor, and antioxidant	Yes	Li et al., 2020
*Euphorbia tirucalli*	光棍树	Steroid	Stems	Treat sexual impotence, warts, epilepsy, toothache, hemorrhoids, and snake bites	Yes	Qiao et al., 2018
*Fagopyrum tataricum*	苦荞	Rutin	Tuberous roots	Reduce cholesterol levels, blood clots, and high blood pressure	Yes	Zhang L. et al., 2017
*Fritillaria hupehensis*	湖北贝母	Fritillaria	Bulbs	Treat hot-type bronchitis with dry cough and heart diseases, antitussive, anti-asthmatic, and expectorant	Yes	Guo K. et al., 2021
*Ganoderma tsugae*	灵芝	Ganoenic acid	Sub-entities	Antioxidant, anti-inflammation, anti-tumor, lipid lowering, and immunity enhancement	Yes	Jiang N. et al., 2021
*Glycyrrhiza uralensis*	甘草	Glycyrrhizin	Rhizomes	Anti-inflammatory, anticancer, antiallergic, and antiviral	Yes	Mochida et al., 2017; Lin et al., 2022
*Gymnema sylvestre*	匙羹藤	Gymnemic acid	Leaves	Treat diabetes, hypolipidemic3, stomachic, diuretic, refrigerant, and astringent properties	Yes	Hossain et al., 2016; Tiwari et al., 2017; Pham et al., 2018; Ayachit et al., 2019
*Gynostemma pentaphyllum*	绞股蓝	Gypenoside	Whole plant	Antitumor, hypoglycemic, hypolipidemic, cardiovascular and cerebrovascular protection, and immunoprotection	Yes	Liang et al., 2019
*Hemerocallis citrina*	黄花菜	Rutin	Flower buds	Relieve depression and promote lactation, and anti-inflammatory	Yes	Liang et al., 2019
*Impatiens balsamina*	凤仙花	2-methoxy-1,4-naphthoquinone	Pods	Treat breast cancer, lung adenocarcinoma, and gastric adenocarcinoma	Yes	Foong et al., 2020
*Lantana camara*	马缨丹	Phenylpropanoid glycosides		Treat cancer, ulcers, asthma, and fever	Yes	Shah et al., 2020
*Lithospermum erythrorhizon*	紫草	Shikonin	Roots	Treat breast cancer and anti-HIV	Yes	Auber et al., 2020
*Macleaya cordata*	博落回	Sanguinarine and chelerythrine	Whole plant	Antimicrobial and animal growth-promoting	Yes	Liu X. et al., 2017
*Magnolia biondii*	望春花	Magnolin and fargesin	Flowers	Anesthesia, anti-inflammatory, analgesic, blood pressure-decreasing, and anti-allergic	Yes	Dong et al., 2021
*Melia toosendan*	川槭	Toosendanin	Fruits	Treat disease caused by a parasite	No	Lian et al., 2020
*Murraya koenigii*	咖喱树	Girinimbine, mahanimbine, murrayafoline, pyrafoline-D, sabinene, α-farnesene, and α-pinene	Leaves and roots	Treat asthma, prostate cancer, obesity,diarrhoea, dysentery, diabetes, and skin eruptions	No	Meena et al., 2017
*Ophiopogon japonicus*	浙麦冬	Methylophiopogonanone A and sprengerinin A	Tubers	Treat cardiovascular disease, cancer, and diabetes	Yes	He et al., 2016; Liu H. et al., 2017; Wu et al., 2019; Fan S. et al., 2020
*Ophiorrhiza pumila*	短小蛇根草	Camptothecin and pumiloside	Roots	Antitumor drug	No	Rai et al., 2021
*Panax ginseng*	人参	Ginsenosides	Roots and rhizomes	Cure central nervous system diseases, chronic diseases, and cancer	Yes	Xu et al., 2017; Xue et al., 2019
*Panax notoginseng*	三七	Ginsinoside and dencichine	Roots	Treat injury-induced trauma and cancer	Yes	Chen et al., 2017; Zhang D. et al., 2017; Fan G. et al., 2020; Zhang et al., 2020; Jiang Z. et al., 2021; Yang Z. et al., 2021
*Papaver somniferum*	罂粟	Morphine and codeine	Fruits	Painkilling drugs	Yes	Guo et al., 2018
*Phellinus gilvus*	桑黄	Agaricic acid, veratrine, and ergosterol	Sub entities	Treat stomachache, inflammation, tumor, and diabetes	Yes	Huo et al., 2020
*Piper nigrum*	黑胡椒	Piperine	Fruits	Treat diabetes and cancer	Yes	Hu et al., 2019
*Platycodon grandiflorus*	桔梗	Platycodin D and E and polygalacin D	Roots	Anti-inflammatory, antiobesity, anticancer, antiviral, and antiallergy	Yes	Kim et al., 2020; Yu H. et al., 2021
*Pogostemon cablin*	广藿香	Patchouli alcohol	Leaves	Remove moisture, prevent vomiting, and stimulate the appetite	Yes	Chen X. et al., 2019
*Pueraria thomsonii*	葛根	Puerarin and daidzein	Roots	Treat influenza, body stiffness, diabetes, and vascular hypertension	Yes	He et al., 2019
*Rehmannia glutinosa*	地黄	Catalpol, rehmaglutosides, and myobontiosideA	Tuberous roots	Strengthen cardiovascular, central nervous, immune, and visceral system	Yes	Ma L. et al., 2021
*Salvia miltiorrhiza*	丹参	Tanshinone IIA, tanshinone I, cryptotanshinone, and 15,16-dihydrotanshinone I	Roots	Treat coronary artery disease and cancer, anti-HIV	Yes	Jia et al., 2019; Guo Y. et al., 2021; Yin et al., 2021
*Salvia officinalis*	鼠尾草	Tujone, camphor, and sugiol	Leaves	Treat choleretic and cancer	Yes	Jantová et al., 2014; Jiang et al., 2017
*Scutellaria baicalensis*	黄芩	Wogonin, baicalein, and baicalin	Roots	Treat liver, lung complaints, and cancer	Yes	Zhao et al., 2019
*Senna tora*	决明	Emodin, glucoaurantio-obtusin, physcion, chrysophanol, gluco-obtusifolin, aurantio-obtusin, obtusifolin, and obtusin	Seeds	Inhibit microbial and parasitic infections. Prevent neurodegenerative diseases and diabetes	No	Kang et al., 2020a,b
*Siraitia grosvenorii*	罗汉果	Mogroside	Fruits	Treat lung congestion, sore throat, and constipation	Yes	Xia et al., 2018
*Sophora flavescens*	苦参	Matrine, oxymatrine, sophorine, oxysophoridine, trifolirhizin, maackiain, kushenol I, kurarinone, and sophorafavanone G	Roots	Antiviral	Yes	He et al., 2015; Wei et al., 2021
*Strobilanthes cusia*	板蓝	Indigo and indirubin	Rhizomesand roots	Treat oral ulcers, skin diseases, influenza A infection, and mumps	Yes	Hu et al., 2021
*Swertia mussotii*	獐牙菜	Secoiridoids, swertiamarin, mangiferin, gentiopicroside, sweroside, oleanolic acid, and xanthones	Whole plant	Treat hepatitis, diabetes, and hyperlipidaemic	Yes	Liu Y. et al., 2017
*Taxus chinensis*	红豆杉	Paclitaxel	Leaves	Anticancer drug	Yes	Xiong et al., 2021
*Trachyspermum ammi*	阿育魏实	Thymol, γ-terpinene, and p-cymene	Seeds	Antifungal, antibacterial, antivirus, and anti-inflammatory	No	Soltani Howyzeh et al., 2018
*Trillium govanianum*	西藏延龄草	Steroidal saponin	Rhizomes	Cure menstrual, dysentery, headache, and fever	No	Singh et al., 2017
*Tripterygium wilfordii*	雷剬藤	Triptolide and celastrol	Leaves	Treat central nervous system diseases and cancer	Yes	Tu et al., 2020
*Zingiber officinale*	生姜	Gingerols	Rhizomes	Inhibit hyperproliferation, inflammation, and carcinogenesis	Yes	Li H. et al., 2021
*Ziziphora bungeana*	白枣	Linarin, cafeic acid, and rosmarinic acid	Whole plant	Treat fever, headache, insomnia, and cardiovascular disease	Yes	He et al., 2020

Regarding future practical applications, on one hand, combining 2nd and 3rd generation sequencing technologies makes full use of the strengths of each, such as long read length, high throughput, and acceptable sequencing costs. On the other hand, single-cell sequencing technology has great application prospects in the mining of active ingredients of Chinese traditional medicinal herbs. Traditional RNA-Seq technologies sequence RNA extracted from a variety of tissues and cells, ignoring intercellular differences. Single-cell RNA sequencing isolates the target cells from the sample and then sequences them, allowing for the unique characteristics of individual cells. Additionally, scRNA-seq has yielded rich results in the fields of tumors, microorganisms, and so on (Li S. et al., 2021). Xu X. et al. (2021) used scRNA-seq to mine trait genes during maize development. It is more accurate and efficient than traditional RNA-seq and uncovers more information.

## Author Contributions

This study was designed by MT and GL. Data analysis was performed by JLL. The figures were organized by YL and HTZ. The manuscript was written by XYL and XG. The manuscript was revised by SQ. All authors made a direct and intellectual contribution to this topic and approved the article for publication.

## Funding

This work was financially supported by grants from the National Natural Science Foundation of China (32002235).

## Conflict of Interest

The authors declare that the research was conducted in the absence of any commercial or financial relationships that could be construed as a potential conflict of interest.

## Publisher’s Note

All claims expressed in this article are solely those of the authors and do not necessarily represent those of their affiliated organizations, or those of the publisher, the editors and the reviewers. Any product that may be evaluated in this article, or claim that may be made by its manufacturer, is not guaranteed or endorsed by the publisher.
