# Supplementary material for: Application of High-Throughput Sequencing on the Chinese Herbal Medicine for the Data-Mining of the Bioactive Compounds
Source: Front Plant Sci. 2022 Jul 14;13:900035. doi: 10.3389/fpls.2022.900035 (PMC9331165; doi:10.3389/fpls.2022.900035)
Supplement: Supplementary file 1 [file Data_Sheet_1.PDF]

Review

Application of High-Throughput Sequencing on the Chinese Herbal Medicine for the data-mining of the Bioactive Compounds

Xiaoyan Liu[1], Junlin Liu[1], Xun Gong[2], Yuzhen Xu[3, *], Min Tang[1, *]

1. *School of Life Sciences, Jiangsu University, Zhenjiang 212013, Jiangsu Province, China*
2. *Affiliated Hospital of Jiangsu University, Zhenjiang 212001, Jiangsu Province, China*
3. *Department of Rehabilitation, The Second Affiliated Hospital of Shandong First Medical University, Taian 271000, Shandong Province, China*

* Corresponding author: Min Tang (mt3138@ujs.edu.cn)

Yuzhen Xu (xuyuzhen@sdfmu.edu.cn)

SUPPLEMENTAL FIGURES

Figure S1 | Chemical structure formula of the bioactive compounds from 11 species.

Areca catechu	*Andrographis paniculate*	*Coptis chinensis*	*Curcuma longa*
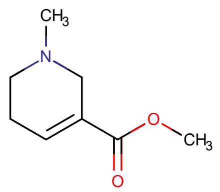	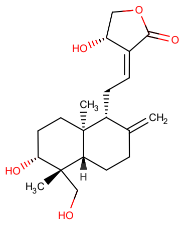	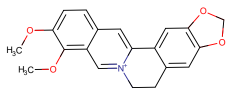	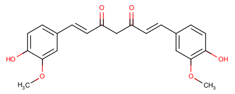
Arecoline	Andrographolide	Berberine	Curcumin
Eucommia ulmoides	***Andrographis paniculata***	***Salvia miltiorrhiza***	***Tripterygium wilfordii***
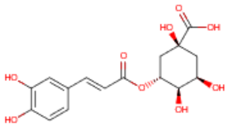	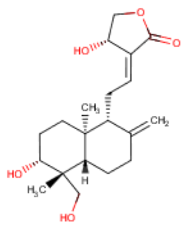	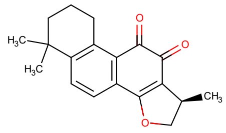	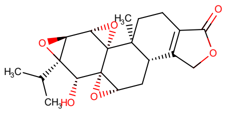
Chlorogenic acid	Andrographolide	Cryptotanshino	Triptolide

Rehmannia glutinosa

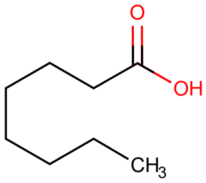

Caprylic acid

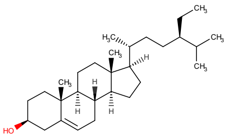

β-sitosterol

Anelica sinensis

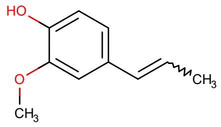

Isoeugenol

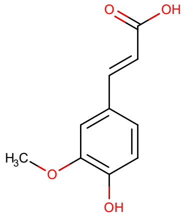

Ferulic acid

Callerya speciosa

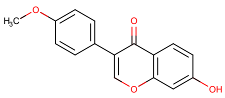

Formononetin

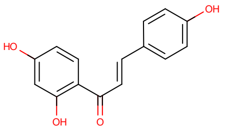

Isoliquiritigenin

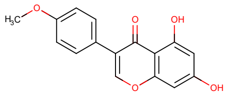

Biochanin A

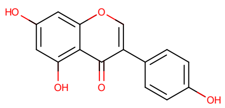

Genistein
